# Activity provider-facilitated patient and public involvement with care home residents

**DOI:** 10.1186/s40900-023-00537-z

**Published:** 2024-01-11

**Authors:** Kerry Micklewright, Anne Killett, Gizdem Akdur, Priti Biswas, Pamela Blades, Lisa Irvine, Liz Jones, Julienne Meyer, Natalie Ravenscroft, Hilary Woodhead, Claire Goodman

**Affiliations:** 1 School of Health Sciences, University of East Anglia, Norwich, UK; 2 NIHR Applied Research Collaboration East of England, Cambridge, UK; 3 Centre for Research in Public Health and Community Care, University of Hertfordshire, Hatfield, UK; 4 Norwich Medical School, University of East Anglia, Norwich, UK; 5 Norwich, UK; 6 National Care Forum, Coventry, UK; 7 National Activity Providers Association, Amersham, Buckinghamshire UK

**Keywords:** Patient and public involvement and engagement (PPIE), Aged, Care home, Activity provider, Older people, Participatory action research

## Abstract

**Background:**

In care home research, residents are rarely included in patient and public involvement and engagement (PPIE) despite their lived experiences of day-to-day care. This paper reports on a novel approach to PPIE, developed in response to Covid-19, and utilised in a large UK-based study focused on care homes. PPIE sessions were facilitated on behalf of the research team by Activity Providers (APs) already working within the care homes. This paper provides an account of how PPIE with care home residents can be achieved.

**Methods:**

An exploratory design was used to see if it was possible to support “in-house” PPIE, with researchers working at a distance in partnership with care home staff. The National Activity Providers Association recruited five APs working in care homes. A series of optional discussion or activity sessions were developed by the research team in partnership with APs, tailored to reflect the research topics of interest and to make sessions accessible to residents with differing needs.

**Results:**

APs facilitated four rounds of PPIE with up to 56 residents per topic, including individuals living with cognitive and communication impairments. Topics discussed included residents’ views on data use, measuring quality of life and the prioritisation of care-related data for study collection. Feedback from the residents was observed to have unexpected and positive changes to participating care homes’ practice. APs valued participation and working with researchers. They identified acquisition of new skills and insights into residents’ thoughts and preferences as direct benefits. Challenges included time pressures on APs and managing emotive feedback. APs were able to approach residents at times convenient to them and in ways that best suited their individual needs. PPIE with residents provided different perspectives, particularly with respect to the importance of different types of data, and constructive challenge about some of the research team’s assumptions.

**Conclusions:**

PPIE with APs as research partners is a promising approach to working in an inclusive and participatory way with care home residents. The voices of older care home residents, including those living with cognitive or communicative impairments, are important for the successful and meaningful completion of research.

**Supplementary Information:**

The online version contains supplementary material available at 10.1186/s40900-023-00537-z.

## Abstract (plain language summary)

In recent years there has been increasing interest in research relating to care homes. It is relatively rare that care home residents are given the chance to influence this research; often, family members or care home staff are asked to speak on their behalf. Research can influence residents’ future care, and it is important to find ways of involving residents in research that are meaningful and enjoyable. This research paper discusses a new approach to involving care home residents in research. It begins by recounting how the approach came about, then covers how well it has worked so far, finally reflecting on the benefits and challenges of working in a new way. The researchers originally planned to go into care homes themselves to speak to residents, but with the onset of the Covid-19 pandemic this was no longer possible. Instead, Activity Providers already working in care homes were recruited via the National Activity Providers Association (NAPA) to help. They used activities and discussion prompts developed with the research team to speak to residents about the study. The research team hoped to make getting involved in research meaningful and interesting for residents. The team also wanted to make sure that as many people as possible living with conditions like dementia could get involved too. Comments and suggestions from residents were fed back to the research team to help them make decisions about how the research should be done.

**Supplementary Information:**

The online version contains supplementary material available at 10.1186/s40900-023-00537-z.

## Introduction

Care home research is complex, crossing disciplines and paradigms, including sociology, gerontology, medicine and health sciences. In the UK the term “care home” refers to all long-term care settings for older people, including those with and without onsite nursing provision. Undertaking research in these environments can be challenging; a considerable number of large UK-based care home intervention studies have produced neutral outcomes [1–6]. Despite over 400,000 older people living in care homes nationally and growth in care home intervention research, relatively few studies provide evidence of care home residents’ inclusion in patient and public involvement and engagement (PPIE) [7–9].

Patient and public involvement and engagement with care home residents can enhance research design and delivery [10, 11]. Without residents’ insights on the day-to-day experience of living in a care home, researchers’ assumptions and questions can go unchallenged. Importantly, without PPIE, residents are unable to contribute their insights, questions or concerns in relation to research with direct relevance to their lives. Residents’ knowledge represents a valuable resource with regards to the epistemic goals of research [12]. Furthermore, it has been argued that exclusion of the knowledge and perspectives of those living or working in care homes in decisions has led to harmful reductionistic approaches to how care should be implemented, impacting upon care quality and the wellbeing of staff and residents [13]. Findings of care home research can carry significant policy implications for the care, and therefore experiences, of residents. Providing opportunities for residents to engage in meaningful and well-designed PPIE is essential to ensure that they can influence the generation of knowledge relating to crucial aspects of their lives. Often, their views are inferred via the voices of others [9, 11, 14].

Studies utilising PPIE with care home residents have historically been small scale and qualitative in design [8]. A recent review found there is growing interest in the role of residents in intervention development and research priority setting [9]. Researchers report a number of barriers to resident inclusion, including: challenges in relation to residents and/or researchers being able to engage meaningfully with the topic and each other; cognitive and physical difficulties and their impacts on the ability to withstand longer sessions; low resident confidence to engage; relationship dynamics (and associated power relations) between residents, staff and relatives; time and resource limitations; challenges linked to care home organisation and structure; and researchers having sufficient flexibility and responsiveness required to facilitate resident involvement [8, 9].

### PPIE in DACHA

The DACHA (*Developing research resources And minimum data set for Care Homes’ Adoption and use)* study is a large scale, complex intervention study that relates to how the collection, recording and sharing of resident data can be optimised to improve care [15, 16]. DACHA study is a National Institute for Health and Care Research (NIHR)-funded collaboration that commenced in 2019 (anticipated completion: May 2024) between nine Higher Education Institutions, a not-for-profit company (the National Care Forum) and a charity (The Health Foundation). The research team consists of 30+ members with various backgrounds, including nursing researchers, care home researchers, geriatricians, health economists, implementation researchers, care sector representatives and PPIE representatives. Within the DACHA study, PPIE is defined as listening to the voices of individuals living, dying, visiting and working in care homes, members of the public and other key stakeholders in the health and social care system to inform the quality of the research and enhance the usefulness of study findings. It is underpinned by a democratic approach, meaning that the groups involved in PPIE contribute to decision making, and by the understanding that to effectively contribute to research PPIE representatives must be treated as respected partners who hold credible knowledge and expertise [17–21]. Importantly, the PPIE team within DACHA (a group of study co-applicants with a specific focus on embedding PPIE activities throughout DACHA, consisting of individuals from both academic and non-academic backgrounds) from the outset aimed to ensure the inclusion of care home residents. The value placed on engagement with residents was based not only on normative values (such as emphasis on empowerment or engagement as an ethical imperative) but also a belief in the unique knowledge held by residents, distinct from the knowledge and perspectives of other involved PPIE representative groups (i.e., family members and/or care providers) [22, 23].

When DACHA was conceptualised, the DACHA PPIE team anticipated face to face engagement (individually and in groups) with residents living in two care homes. The Covid-19 pandemic and subsequent strict visiting restrictions meant a different approach was required. Care home providers, managers and staff were overwhelmed with the demands of keeping people safe and responding to pandemic guidelines, reducing their capacity to engage with researchers. A solution could be for in-house care home staff to facilitate PPIE on behalf of the research team. Activity Providers (APs), also known as ‘Wellbeing Leads’ or ‘Activity Coordinators’, are individuals employed by care homes to plan and facilitate meaningful activities with residents to support their mental and physical wellbeing. Generally, a formal qualification is not required to become an AP, though completion of the Care Certificate and an accredited learning pathway is encouraged [24]. Throughout the pandemic, as staff members, they could approach and engage residents where the DACHA PPIE team could not.

There were anticipated benefits from partnering with APs. APs are already embedded within the care home setting, holding an implicit understanding of care home routine, resident preferences and resident needs, including how to maximise individual residents’ ability to communicate and engage with sessions. Individuals embedded in a care home could potentially have greater flexibility in relation to session timings, being based onsite throughout their working day. APs have pre-established relationships and residents may feel more comfortable sharing their thoughts (or a wish to decline participation) than with unknown researchers. Given the importance of rapport, relationship building and trust in PPIE, the team considered that this could be an important advantage [25]. APs also possess a skillset that would help to translate discussion topics arising from DACHA into meaningful activity sessions for care home residents.

Undertaking PPIE by proxy had risks for the team. Participating APs were not familiar with the process of completing research generally (and DACHA study specifically). This raised questions about how we would communicate the values and goals of PPIE to residents. Resident feedback would also be via APs, and how APs elicited, interpreted and recorded resident participation and feedback would ultimately influence what information the DACHA research team received.

This paper provides an account of how PPIE with care home residents was facilitated in DACHA study with the purpose of demonstrating the process, challenges and potential for expanding this approach with groups who historically have had limited representations in PPIE work.

## Methods

The approach was exploratory, asking if PPIE by proxy in long term care settings was appropriate and desirable.

### Recruitment of Activity Providers

A member of the study’s PPIE panel of care providers and family members suggested APs could facilitate resident involvement. The DACHA team had existing links (through JM) with the National Activity Providers Association (NAPA). NAPA is a charity that advocates for the promotion of activity and engagement and offers training, resources and professional development for APs [26]. Partnering with NAPA facilitated recruitment and support of APs throughout; it also avoided burdening care home managers with the task of liaising with researchers.

The DACHA PPIE team, NAPA CEO and NAPA Wellbeing Support Manager met online and developed a protocol. Members of the DACHA PPIE team presented at the Activity Providers’ Advisory Group in May 2021, giving an overview of the study, types of public involvement and the proposed collaboration with NAPA. Information about the study was then sent to NAPA members in June 2021. Interested APs based in care homes in England that catered for older (65+) permanent (i.e., non-respite) residents, were invited to respond to the NAPA Wellbeing Support Manager. Six APs expressed an interest, with two meeting the DACHA team and agreeing to take part. These two APs eventually had to step away from their roles, so the process was repeated and three further APs were recruited and joined the project in July 2022. Recruited APs worked in a range of settings (see Table 1). All participating APs had no prior research experience and each AP was employed at a different care home. Details regarding the characteristics of participating APs are shown in Table 2.

**Table 1:** Care homes participating in DACHA study PPIE with residents

Type of care home	Number of residents	Area of specialism
Residential and nursing care	57	Older age and people living with dementia
Nursing	27	Older age and people living with dementia
Dementia care village	72	Older age and people living with dementia
Residential and nursing care	49	All ages, including people 65+ and those living with dementia and neurological issues
Care home and neurological rehabilitation centre	82	All ages, including people 65+ and those living with dementia and neurological issues

**Table 2:** Characteristics of participating activity providers (Note: pseudonyms used)

Activity provider	Gender	Job title	Years of experience as an AP (At time of recruitment)
Anna	F	Activities/Events Co-ordinator and Community Engagement Officer	Less than 1 year
Emma	F	Activities Co-ordinator	1–5 years
Teresa	F	Experience Co-ordinator	1–5 years
David	M	Lifestyle and Wellbeing Lead	5–10 years
Steven	M	Lifestyle and Events Manager	5–10 years

APs were reimbursed for their time via bank transfer (£20.22/h) or high street vouchers (£20.00/h). APs were offered four hours reimbursement per topic to cover preparatory and feedback sessions and administrative tasks. APs were also offered statements and certificates for their Continuing Professional Development (CPD) portfolios to evidence their participation. NAPA was renumerated for their facilitation and support of the project (£350 per annum).

### PPIE process

#### Topic selection

The DACHA PPIE team (AK/KM) liaised with researchers leading DACHA study’s work packages to determine the most relevant and timely topics and questions to explore with residents; for example, a topic exploring residents’ understanding of ‘quality of life’ was selected in relation to quality of life outcome measurement selection [27]. Prioritisation was given to topics and questions where resident feedback would have the greatest influence on the research process. An overview of the PPIE process is provided in Additional file 1: Figure S1. A list of topics explored with residents is presented in Additional file 2: Table S1.

#### Activity development

Once a topic was selected, the PPIE team (KM) prepared potential activities to explore the topic with residents; this process arose from discussion with recruited APs. To minimise additional work for APs, the PPIE team (AK/KM) were responsible for suggesting activities and preparing resource packs to stimulate engagement with residents about DACHA study. Draft activities were discussed with APs at online preparatory meetings, where feedback regarding suggested amendments to wording, content and potential methods of activity facilitation were discussed. There was also opportunity to raise questions or concerns. Following AP feedback, revised resource packs were sent out to APs in digital or paper format, depending on AP preference. Residents were not directly involved in this process, though their feedback to APs about participation (for example, if they had particularly enjoyed an activity or wished to engage in a different way next time) was fed into the development of future activities.

A range of activities were included in resource packs, including structured discussions (for example, a series of talking points or discussion of a theoretical scenario), creative sessions (including art, photography, creative writing, cooking group or music sessions) and questionnaires that could be completed independently or incorporated into a facilitated discussion. These activities were offered to residents as an optional activity in addition to their usual care. For residents living with cognitive or communicative difficulties, resource packs included prompt cards with visual cues and easy read resources. Printable information leaflets about the DACHA study were included for interested residents.

#### Activity implementation

Activity Providers had a period of between two and four weeks to complete sessions with residents. APs were encouraged to modify session facilitation with respect to resident needs and interests, including if residents preferred one-on-one or group participation. There were no recruitment targets or limitations imposed regarding the inclusion of residents with cognitive, communicative or mobility difficulties. Prior to commencing an activity or discussion, APs approached residents about DACHA study and the potential to participate in PPIE. Residents were then able to agree or decline to take part on the understanding that no potentially identifying information about them would be shared with the research team. APs were encouraged to build activities into their usual schedule where this was not disruptive for residents (for example, discussing the topic of interest at weekly resident meetings or incorporation into pre-existing creative group sessions).

APs kept written notes of views, questions and comments from residents and about their own experiences. They reported on: session content, including resident feedback, questionnaire responses and creative outputs; resident thoughts on the topics’ relevance; the extent to which residents engaged with sessions (both in terms of numbers participating and perceived interest within sessions); barriers to engagement; AP experiences regarding participation and facilitation, including usability/helpfulness of the resources; suggestions or concerns; and the number and observed experiences of participating residents with cognitive or communication difficulties. All feedback from and regarding residents was anonymous. APs later fed back their findings to the PPIE team (AK/KM) at a subsequent recorded online debrief meeting.

The PPIE team were aware of the potential for APs to inadvertently report their own beliefs and interpretations rather than residents’ views. To minimise this, the PPIE team sought to maintain a focus on what residents had done or said when participating in activities, using questions and prompts during debrief sessions to clarify what residents had expressed (e.g., “What made you note that?”, “Did they [the resident] express that directly?”). After each debrief session, the PPIE team reflected on any areas where it was harder to discern residents’ views from the feedback received and any required amendments to how debrief sessions were facilitated.

Support to APs was offered via the NAPA Wellbeing Support Manager, who is particularly experienced in advising members on professional development, service development and AP wellbeing. The Wellbeing Support Manager provided advice, troubleshooting and acted as an individual independent of the PPIE team that APs could approach with any concerns. They attended the preparatory and wrap-up meetings (n = 6/8). Both the Wellbeing Support Manager and the PPIE team were available via email or online meetings for questions or concerns throughout the activity facilitation periods. Any potential safeguarding concerns arising from sessions were addressed following the procedures of the care settings APs were based in.

### Capture and communication of feedback

Detailed minutes summarising recorded wrap-up meetings were created by a member of the PPIE team and circulated to allow APs to check that resident feedback had been appropriately captured and meanings correctly interpreted. Any suggested amendments were addressed and minutes were then shared with the wider DACHA study team. Minutes, as opposed to full transcripts, were chosen as a succinct way of communicating key feedback to researchers, though recordings of wrap-up meetings were kept for the duration of the study (with the permission of meeting attendees) in case questions later arose that required review of the original discussion content. Minutes did not undergo a formal analysis process by the PPIE team to avoid resident feedback undergoing further interpretation (and potential changes to intended meaning) before reaching DACHA researchers.

Verbal feedback about how the residents’ views and responses were incorporated into the research process (or if they were unable to be actioned) were shared with APs at subsequent preparatory and wrap-up meetings. APs were asked to share this with residents [28].

### Impact and evaluation

Information regarding impact (defined here as “the changes, benefits and learning gained from the insights and experiences of…the public”) was logged in a shared document by a member of the PPIE team (KM) as the collaboration progressed [29]. Information was drawn from meeting minutes and agendas (meetings between the PPIE team and APs, DACHA study research management meetings and PPIE team meetings), a PPIE team worklog, verbal and written feedback from DACHA researchers and a reflective workshop session completed as part of a research team residential. Data was sorted in relation to PPIE stakeholder group and then study work package to help establish how feedback from different stakeholders influenced specific aspects of DACHA study. The PPIE team drew upon principles of thematic content analysis to identify agreement and divergence in feedback, which aspects of the study were influenced by PPIE stakeholders to a greater or lesser extent and to explore perceived strengths and weaknesses in both the approach to PPIE with residents and DACHA study’s overall PPIE strategy [30].

Formal evaluation of this approach to PPIE with residents (as well as the overall study PPIE strategy) continues, and a planned follow-on study has been funded to further investigate the potential of this approach [31]. AP-facilitated PPIE with residents will be used to inform three different care home studies, creating three ethnographic case studies. Resident PPIE sessions will be observed and recorded by a researcher unaffiliated with participating studies. To understand perspectives on the meaningfulness and burden of this approach, interviews with residents (including those that did not participate in PPIE), residents’ family members, care home staff and care home managers will be completed. Evaluation will include cost analysis, utilising the framework suggested by Pizzo et al. [32].

## Results

### Resident participation

#### Activities completed

Residents completed PPIE sessions with APs for four different topics related to the DACHA study—see Additional file 2: Table S1. Between 14 and 54 residents participated per topic. The fourth topic saw a lower number of residents involved due to a Covid-19 outbreak at a participating care home.

The importance of an approach tailored to individual residents was evident throughout, and resident preference in relation to potential activity formats could not be assumed; for example, the PPIE team did not initially include questionnaires in the activity kits with the expectation that residents may find them less engaging. However, APs fed back that some residents expressed a preference for a questionnaire format that would allow them to work methodically through and reflect on the questions being asked. Questionnaires were subsequently added to all later activity kits (and successfully used with residents) as a supplementary resource that invited thoughts on the topic of interest.

Activity Providers were flexible in how they completed sessions. Some slotted activities into pre-existing resident groups (for example, weekly creative or “current events” sessions), while others (n = 3) arranged dedicated discussion or more formal consultation sessions in response to resident preferences. APs also completed one-to-one sessions with individuals who they knew were unlikely to attend groups. APs demonstrated creativity in their approach to engaging residents living with a range of needs: ‘We’ve got a lady who is blind—for her we used…sensory tactile pads…we had pots of different smells…we used crafts and reminiscence products [to explore the topic of interest, quality of life]’ (David, AP).

APs had to exercise their judgement with respect to activity ideas or topics that specific residents may find distressing or difficult to engage with, sometimes omitting certain elements of the activity pack; one example included an AP who avoided using sorting cards or discussion questions relating to medication management, as they had previously found that this could be a particularly anxiety-provoking topic for some residents. In these cases, APs were presented with an ethical dilemma, weighing up a desire to provide a resident the opportunity to contribute their views with a wish to minimise their distress. APs reported that these residents were offered opportunities to engage with other activities or discussion questions.

Reported barriers to resident engagement included greater difficulty when attempting to include newer residents with whom APs were less familiar and attempting to complete activities when both care homes (and APs specifically) are busier, noting December and January to be a challenging time to fit in PPIE sessions. Engaging residents living with cognitive impairments in a meaningful way was reported to be more challenging, with APs taking different approaches to navigating this. One AP completed 1:1 sessions with these residents, feeling that it maximised the ability of residents to participate, while another split up an activity in short sessions over several days.

#### Feedback about participation

The APs focused on the strength and level of participation as indicators of successful engagement. Positive impacts for residents who participated were framed as giving residents opportunities to speak and make an active contribution: *‘…once again, they felt empowered by doing this…’* (Anna, AP); *‘…residents felt valued, present, involved and listened to.’* (Emma, AP). Residents’ confidence to contribute was shared as evidence of interest and willingness to engage: *‘I didn’t have to force anything. People were happy to speak up.*’ (Teresa, AP). APs reflected that completing the sessions gave a forum for residents to discuss elements of their lives or care that would perhaps not be revealed otherwise: *‘…I thought, “Right…” Because before they [the residents] wouldn’t have mentioned it, but by asking a few simple questions…’* (Teresa, AP). This had unexpected consequences where APs sometimes fed back that they had successfully actioned resident suggestions and requests that had arisen via the previous round of resident PPIE. For example, one care home changed the process by which residents were made aware of upcoming healthcare appointments following discussion that this was not always clear, while another care home arranged for a resident to visit a destination with personal importance that the resident was keen to attend (but that their care home was not previously aware of) in response to a PPIE activity relating to quality of life. As members of staff, APs felt a responsibility to action changes in response to feedback: *‘It made me quickly go straight to my manager and say, “We have to do more, we have to do this, this and this”.’* (Emma, AP).

### Feedback from APs

Feedback from APs about (a) participating in the project, (b) resource pack contents and (c) facilitating sessions with residents was almost uniformly positive. They described the activities as helpful, easy to use and accessible to residents, including those living with dementia. APs appeared to enjoy taking part in research: *‘It added life to sessions. It was something new.’* (Emma, AP). APs felt the work held value and that topics discussed were relevant to both their current and ongoing practice: *‘This sort of project, I think this should be in every day [practice]…’* (Teresa, AP). Participation was represented as an opportunity for professional development and a platform for gaining skills, new insights and trying new approaches to engaging with residents: *‘It was useful to do…I found out lots of things…you don’t always think about these things in routine [practice].’* (Emma, AP). However, hearing resident feedback sometimes generated additional work for APs in actioning feedback or addressing any concerns voiced. Given their role within the care home, APs felt a responsibility to meaningfully address resident suggestions and explore potential changes to care delivery in a way that would not be expected of visiting researchers.

Additionally, while the PPIE team and Wellbeing Support Manager had discussed with APs the potential for feedback to be negative and how they might respond to this, discussions could still sometimes be surprising or upsetting. Following completion of activities that explored resident quality of life (see Additional file 2: Table S1), one AP reflected on an encounter with a resident who no longer felt that the concept of quality of life applied to them. It was suspected that this view arose from a perceived irrevocable loss of autonomy and identity associated with transitioning to life in a care home. Given the role of APs in promoting wellbeing and positive experiences within care home environments, the AP found this upsetting. However, having these conversations (and the opportunity to begin exploring and responding to these feelings with residents) was seen as valuable: *‘Obviously it’s very emotionally straining, listening, but I can step away from that and think, “at least they’ve opened up”. It’s difficult, but there’s always a positive element.’* (Teresa, AP). These findings highlight the importance of supervision and debriefing to support APs and ensure issues raised are addressed.

### Feedback from NAPA

Feedback from NAPA was sought throughout the collaboration. Involvement in DACHA study as facilitators of PPIE aligned with the skillset and relationships APs possess. How APs had embraced creative ways to engage with residents was felt by NAPA representatives to validate APs’ skills in a sector where their contribution could be overlooked. Participation was viewed by NAPA to have yielded positive outcomes for residents, their members and care homes. NAPA were interested in promoting further cross-disciplinary research and study collaboration.

### Learning from and responding to resident feedback

The DACHA research team received resident feedback relevant to the study after completion of each activity topic. This feedback helped to clarify thinking, influenced decision making and enabled the team to better understand how data collection and sharing could impact on care for residents. Feedback was particularly valuable in relation to prioritising types of resident data to be collected by DACHA study (see Additional file 2: Table S1). DACHA study explores collecting and linking together resident data between care providers (such as care homes, GP surgeries and hospitals) to optimise care, minimise duplication and reduce the risk of incorrect or outdated information being held about residents, among other potential benefits [15, 16]. The research team reflected that guidance from PPIE stakeholders, including residents, would be helpful in identifying the most important data to target for collection as part of DACHA study. Resident feedback influenced the DACHA research team’s perceived importance of some data categories. For example, data relating to certain types of healthcare utilisation, including the frequency and nature of visiting healthcare professionals (such as district nurses and community allied health professionals), was initially perceived to be a low priority for capture. However, feedback from APs suggested that residents felt strongly these data represented how residents are supported by external services and should be a priority for capture. Consequently, this data was re-ranked as high priority and incorporated in the planned data collection. Additionally, care home residents (alongside other public involvement stakeholders) advocated for the importance of capturing comparative data concerning resident care-related quality of life, which prompted extensive work on the part of the DACHA team to identify appropriate outcome measures to achieve this [27].

The research team were interested to note instances of agreement and divergence between feedback from residents and other public involvement stakeholders (such as care home staff or family members), reinforcing the unique voices residents possess and the importance and value of collaborating with them. One example demonstrating a difference in priorities was in relation to a proposed analysis plan for the secondary analysis of a pool of anonymised resident data. PPIE stakeholders were asked what they would most want to learn from data. Where care providers and family members focused on analysis to aid constructive benchmarking of care homes, residents’ interests appeared to lie predominantly in using analysis to explore the impacts of hospital admissions on resident health, wellbeing and function. In this case, both priorities could be incorporated in the analysis plan. Where divergence between feedback could not be fully accommodated, the DACHA research team discussed options within the team (and sometimes with PPIE stakeholders, including residents), to reach agreement.

The DACHA team were not always able to action responses to resident feedback. Residents wanted real-time data sharing of the information collected about residents by the DACHA study, emphasizing the importance of up-to-date care information being accessible to both themselves and, where appropriate, their next of kin. This fell beyond the remit of the DACHA study, though the research team reflected that this feedback was useful to help (a) anticipate direction of travel in relation to how resident data might be used in future practice and (b) shape recommendations for subsequent work.

The sessions inevitably generated feedback that was not always relevant to the study questions or beyond the scope of DACHA study. However, as previously mentioned, resident feedback sometimes prompted changes in practice within participating care homes. Residents at one care home voiced strong wishes to be more involved in their care planning and to be able to regularly review plans with their care team, which was fed back to senior staff and consequently incorporated into routine care. The PPIE team reflected upon the value of PPIE activities that support a culture change normalising resident engagement and influence on practice. While not directly conferring knowledge to DACHA, this was a sign of reciprocity and the mutual benefit of participation.

## Discussion

### Involving residents

This paper reports on a novel approach to PPIE with older people that successfully involved residents from five care homes in research. Their participation informed key study processes, helped to guide future research and triggered changes in practice within the care home setting. This approach to PPIE shows potential to deliver a democratic and empowering approach to PPIE for use in future research projects [19].

Successful participation of older care home residents in PPIE activities over the course of multiple topics is an important finding in itself. Care home residents are often excluded from being contributors to research that directly involves them; this includes studies where PPIE is otherwise well-considered and integrated into research [33, 34]. One recent example includes seeking the views of staff, but not residents, when developing research priorities relating to care homes for older people [35]. The authors reflect that there may be ways of overcoming these challenges in future [36]. Care home residents are often living with complex physical and cognitive needs [37]. This can reduce expectations about what is possible and present barriers (perceived or actual) that requires PPIE approaches to move beyond conventional methods [38–40]. This paper reflects an approach that is based on resident ability and empowerment (as opposed to assumption of loss of ability and voice).

### Facilitation by APs instead of trained researchers

The delivery of sessions by APs as opposed to trained researchers is a form of proxy working which we do not believe has been attempted previously within this setting. This appeared in large part to confer advantages, though there were also limitations. In terms of advantages, APs could capitalise on their in-depth knowledge of residents. Being onsite and knowledgeable about the care home routine, APs were able weave sessions into pre-existing schedules and had flexibility in terms of session timings, which can be important for accommodating residents living with fatigue or other health considerations [8, 9]. APs demonstrated skill, flexibility and creativity in working with the PPIE team to translate technical research topics into activities that residents could meaningfully engage with and in tailoring sessions to maximise resident engagement. Despite not being given targets, APs achieved the involvement of high numbers of residents—up to 54 for one topic—demonstrating the potential of this approach to offer researchers insights from a range of individuals who may have different life experiences and perceptions.

The experience, curiosity and enthusiasm of the APs, with the support of their member organisation, were integral to the success of this approach to PPIE. It should not be assumed that all APs would feel able to incorporate PPIE activities into their practice; this was a negotiated process, based on partnership working. The importance of positive collaborative working resonated throughout the process and was essential for success. This was evident when co-designing activities, where AP feedback about wording and presentation helped circumvent potential issues hitherto unanticipated by the DACHA PPIE team. APs involved in the project not only came from different care settings (in terms of set up and resident needs) but also different stages in their careers, with varying levels of experience. There was no evidence to suggest that the inclusion of less experienced APs led to a lower level of engagement. The PPIE team noted the additional value participation was perceived to confer in terms of (a) skills development and (b) the chance to exchange ideas with other participating APs (for example, at debrief sessions) for these APs. It is possible that being an AP is an isolated role and one of the unanticipated benefits was the opportunity to pool learning and experiences.

The PPIE team were entirely reliant on the feedback of APs. It is possible some resident feedback, including negative assessments, were missed. APs appeared to have pre-existing positive relationships with residents, which smoothed the way for participation; this was a self-selecting group. As such, it is reasonable to hypothesise that (a) APs who have positive relationships with residents will be more interested in engaging in PPIE and (b) residents who viewed APs positively are more likely to have agreed to involvement. The positive resident feedback about the process or life in a care home may reflect pre-existing reciprocity and shared appreciation. However, the finding of constructive but critical resident feedback for each topic and the effort of APs to involve residents who did not usually engage in scheduled activities suggests these characteristics were not a prerequisite. We were not able to establish how many residents declined to participate or why, though we intend to explore this in future work evaluating this PPIE approach [31].

As previously discussed, APs exercised their judgement with regards to topics or questions that may provoke distress for individual residents, tending at times to avoid these during PPIE sessions. Given their role in maintaining resident wellbeing, that residents may be vulnerable, that activities were intended to be enjoyable and that any distress arising from activities would then need to be addressed by the AP, this is understandable. However, it could be argued that understanding the causes of this distress may be important with respect to PPIE and that a trained researcher may have felt able to explore this in a way that an AP would not.

### Inclusivity and generalisability

This PPIE approach enhanced the geographical spread of care home residents who participated. Had the DACHA PPIE team been able to utilise their initial plans, all residents would have been based within one county local to the PPIE team; by recruiting APs through NAPA, the care homes participating were based in five different counties, facilitating greater inclusivity and potential breadth of resident life experiences and backgrounds. This being said, as resident demographic information was not shared with the PPIE team, the level of diversity with respect to factors such as ethnicity, gender and socioeconomic status among participating residents was unclear. Formal evaluation of this AP-facilitated PPIE approach will include cost-analysis and further examination of equality, diversity and inclusivity [31].

### Methodological limitations

This approach was not without its challenges and limitations. Despite efforts to incorporate PPIE sessions into routine AP work, there were increased workload and time pressures. The two APs initially recruited were also not retained throughout, having transitioned to new roles. Recruitment and support of APs was resource intensive, and continuity was lost with those who left the project. However, recruitment of new APs was successful and all APs spoke positively about their involvement in DACHA PPIE activities. The second round of recruitment benefitted from earlier learning and afforded new perspectives from APs (in relation to potential methods of activity delivery) and residents (in terms of lived experience and insights).

The development of activities was challenging for the DACHA PPIE team, who had to create resources for residents they had never met using the guidance of the APs. The PPIE team noted a tension between maintaining fidelity when translating research topics into activities and in keeping activities meaningful for residents who were not always able (or interested) in engaging with the more complex elements of DACHA study. This applied for more technical topics such as data analysis, where the activity pack required several iterations. Additionally, although resident feedback was considered when developing subsequent packs, residents were not directly involved in topic selection and activity preparation and as such this may have compromised the relevance and effectiveness of activities. Given that, post-pandemic, direct collaboration with residents to develop activity packs may be possible, exploring residents’ interest in doing so may be a beneficial avenue to explore.

An important consideration in relation to this approach is the potential influence of receiving feedback via APs (as opposed to directly from residents). As previously discussed, this approach was a response to unanticipated limitations imposed by the Covid-19 pandemic, meaning that a level of pragmatism was required. However, this does not diminish the potential for residents’ views to be inadvertently misunderstood or misrepresented when ‘filtered through’ another individual, thus impacting upon the knowledge and perspectives generated via this approach and its effectiveness as a form of PPIE. Life experiences, perceptions of the elderly and the unique professional role held by APs may influence how they understand the words and actions of residents. The PPIE team attempted to discern direct report of residents’ thoughts from interpretation, though the extent to which this was successful will require exploration in future research. It may be that development of training for APs with regards the goals and purpose of PPIE and the importance of reflexivity would be a beneficial and supportive way to address this issue.

As previously noted, discussions at times diverged from the topic at hand or were interpreted in a way (either by APs or residents) that meant feedback was not directly applicable to DACHA research processes. There were tensions with feedback that was specific to the care home and future work should consider how PPIE could become a direct commentary on the performance of a single setting. Despite this, much of the feedback returned by APs held relevance to DACHA and could be utilised to influence the research process. The detailed accounts of care home life offered a helpful level of granularity and detail that aided the research team’s understanding of how their work could influence the day-to-day functioning of care homes and residents’ experiences.

While the geographical spread of participating care homes was advantageous, given that a limited number of residents from five care homes contributed, we are mindful that we cannot be certain that views expressed are representative of all care home residents.

Lastly, though the approach aligns with the aims of PPIE in DACHA study and has appeared to beneficially influence research processes and decision-making, the extent to which it enhanced research quality and the usefulness of study findings will not be evident until study completion.

### Future work and evaluation

This approach is not aligned with an established theoretical or conceptual framework. This was, in large part, due to the approach being emergent in response to the Covid-19 pandemic. The use of frameworks is encouraged to improve the quality of PPIE; as such, this could be considered a limitation of this approach [41]. Previous research indicates that established frameworks to support PPIE are often not utilised beyond the group that developed them, further suggesting that building bespoke frameworks specific to the situation may be more effective [21]. Further funded study is planned to evaluate this approach and, building upon this, development of a framework to support it may be a beneficial way of strengthening use of this approach in future.

The PPIE resources created could be developed and refined in future studies both for use with individuals living in care homes and other groups. Further research is underway alongside exploration of the views of wider stakeholders (including non-participating residents, family members, care home staff and care home managers) to capture impacts [31].

## Conclusion

Facilitation of PPIE by APs (as part of a wider PPIE approach) successfully resulted in tangible positive impacts in terms of influence on DACHA study research processes and resident care [4]. This approach presents a promising way through which older people living in care homes could be included in empowering and mutually beneficial PPIE, ensuring that they are acknowledged as important stakeholders in the production of knowledge that holds direct relevance to, and implications for, their lives. It also represents a way through which APs, a skilled group whose expertise is under-utilised in research, can further develop and engage as a meaningful part of the research process. Further research utilising an ethnographic methodology is planned to explore the full potential of this approach in future studies [31].

### Supplementary Information

**Additional file 1: Fig. S1.** A summary of the PPIE process, with activity providers facilitating the inclusion of care home residents.

**Additional file 2: Table S1.** An overview of PPIE topics explored with care home residents.

## Data Availability

Data and materials generated from this work (specifically, minutes reporting anonymous resident PPIE feedback and activity materials) are available from the corresponding author on reasonable request. Data and materials from the wider DACHA study are available elsewhere (please see https://dachastudy.com/dacha-outputs/).
