# Supplementary material for: Activity provider-facilitated patient and public involvement with care home residents
Source: Res Involv Engagem. 2024 Jan 11;10:7. doi: 10.1186/s40900-023-00537-z (PMC10782785; doi:10.1186/s40900-023-00537-z)

Additional file 1: Fig. S1. A summary of the PPIE process, with activity providers facilitating the inclusion of care home residents.


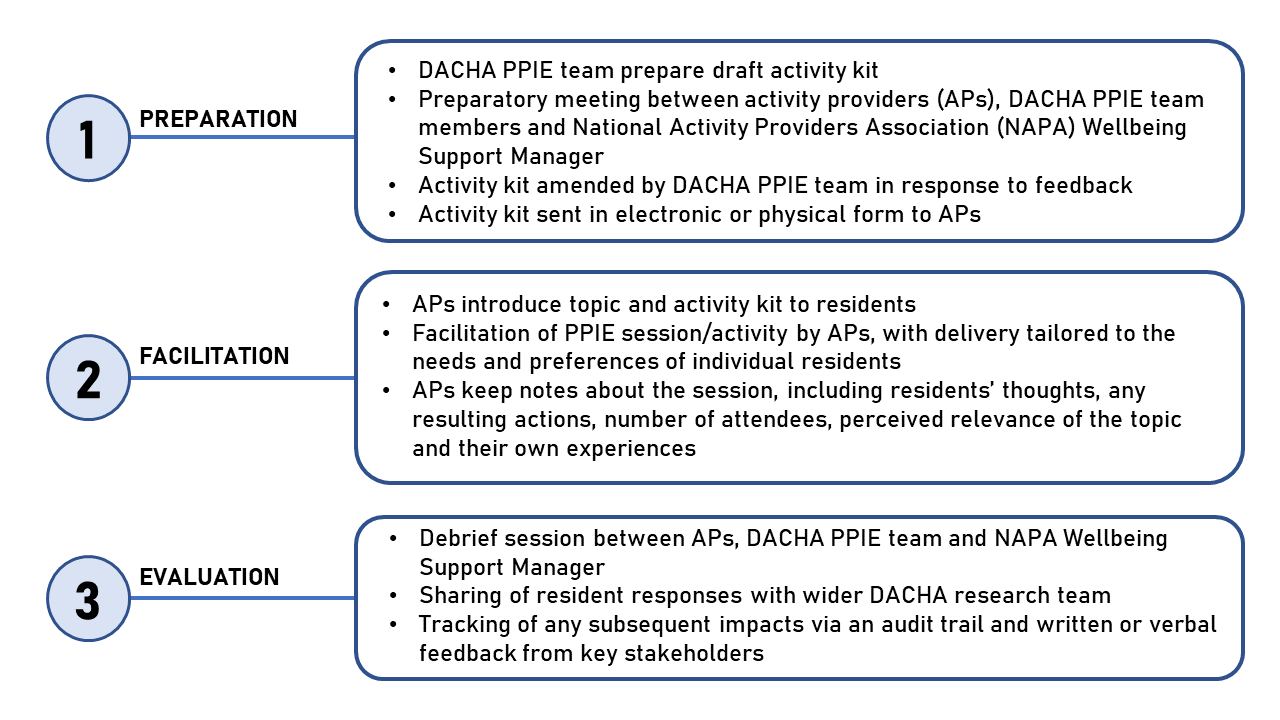

Supplement: Supplementary file 1 — Additional file 1: Fig. S1. A summary of the PPIE process, with activity providers facilitating the inclusion of care home residents. [file 40900_2023_537_MOESM1_ESM.docx]
