# Supplementary material for: Activity provider-facilitated patient and public involvement with care home residents
Source: Res Involv Engagem. 2024 Jan 11;10:7. doi: 10.1186/s40900-023-00537-z (PMC10782785; doi:10.1186/s40900-023-00537-z)
Supplement: Supplementary file 2 — Additional file 2: Table S1. An overview of PPIE topics explored with care home residents. [file 40900_2023_537_MOESM2_ESM.docx]

Additional file 2: Table S1. An overview of PPIE topics explored with care home residents.

| **Topic** | **PPIE session timings** | **Number of APs facilitating sessions/engaged with DACHA study** | **Reported number of residents involved** |
| --- | --- | --- | --- |
| **Information sharing between care settings**  *Various health and social care providers hold data about care home residents. This topic provided an explanation of what DACHA study is to help residents understand the purpose of PPIE and feel able to contribute. Activities and discussion then explored what data residents think care providers should hold, what should be shared between providers and what data residents feel is more sensitive. This topic helped to introduce the idea of data sharing and explored how residents feel about this, as the collection and sharing of resident data is central to DACHA study.* | September 2021 | 2 | 47 |
| **Information for inclusion in care plans**  *DACHA study aims to clarify the types of data that should be collected about care home residents nationally, bringing this together in a prototype Minimum Data Set (MDS). This PPIE topic explored the idea of a fictitious person who is moving into a residential home. Care home residents were asked about what sort of information should be included in this resident’s care plan to enable the best care. Residents were asked about what types of information they perceived to be more or less important and who might be able to tell care homes this information. This helped inform a) what data should be collected about care home residents by DACHA study as part of the prototype MDS and b) whether the DACHA study team had missed any potentially relevant care providers who may hold important data about residents.* | January 2022 | 2 | 54 |
| **Care-related quality of life**  *The DACHA study team received strong feedback (from both residents participating in the previous PPIE topic and other study stakeholders, including care providers and family members) that resident quality of life data should be collected as part of DACHA study’s prototype MDS. However, it is rarely captured in routine data collection and a way of doing so would therefore need to be identified and agreed upon. Conceptualising quality of life is challenging. This topic aimed to help clarify what residents understand the term “quality of life” to mean and what quality of life encompasses to help inform how this could be meaningfully captured by care homes and which outcome measures might best facilitate this.* | August 2022 | 3 | 45 |
| **Priorities for data analysis**  *One of the work packages in DACHA study relates to linking together care home resident data gathered from different health and social care providers. Pooling this data presents opportunities for secondary data analysis and learning about the experience of living in care homes. Residents were asked what learning is most important to them with regards to improving health and wellbeing for care home residents, linking back to the types of data available for analysis. Their responses were used to help inform what analyses were completed.* | January 2023 | 2 | 14 |
